# Ethyl (*Z*)-2-(4-chloro­benzyl­idene)-3-oxobutano­ate

**DOI:** 10.1107/S1600536811000717

**Published:** 2011-01-12

**Authors:** Shaaban K. Mohamed, Antar A. Abdelhamid, Atash V. Gurbanov, A. M. Maharramov, Seik Weng Ng

**Affiliations:** a School of Biology, Chemistry and Material Science, Manchester Metropolitan University, Manchester, England; b Department of Organic Chemistry, Baku State University, Baku, Azerbaijan; c Department of Chemistry, University of Malaya, 50603 Kuala Lumpur, Malaysia

## Abstract

The C=C double-bond in the title compound, C_13_H_13_ClO_3_, has a *Z* configuration. The aliphatic substituents at one end of the double bond, *i.e.* the CH_3_CO– and C_2_H_5_O_2_C– groups, are aligned at 82.1 (3)° with respect to each other.

## Related literature

For related structures, see: Deng *et al.* (2007 ▶); Shi (2008 ▶).
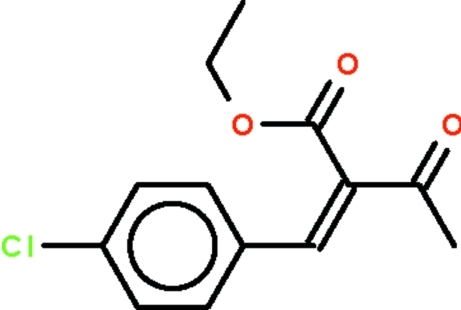


## Experimental

### 

#### Crystal data

C_13_H_13_ClO_3_
*M* *_r_* = 252.68
Monoclinic, 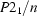

*a* = 9.9956 (6) Å
*b* = 7.7487 (5) Å
*c* = 16.2709 (10) Å
β = 99.624 (1)°
*V* = 1242.49 (13) Å^3^
*Z* = 4
Mo *K*α radiation
μ = 0.30 mm^−1^
*T* = 295 K
0.20 × 0.20 × 0.20 mm

#### Data collection

Bruker APEXII diffractometer
Absorption correction: multi-scan (*SADABS*; Sheldrick, 1996 ▶) *T*_min_ = 0.942, *T*_max_ = 0.942
11255 measured reflections
2790 independent reflections
1968 reflections with *I* > 2σ(*I*)
*R*_int_ = 0.069

#### Refinement

*R*[*F* ^2^ > 2σ(*F* ^2^)] = 0.065
*wR*(*F* ^2^) = 0.201
*S* = 1.02
2790 reflections
156 parameters
H-atom parameters constrained
Δρ_max_ = 0.40 e Å^−3^
Δρ_min_ = −0.52 e Å^−3^

### 

Data collection: *APEX2* (Bruker, 2005 ▶); cell refinement: *SAINT* (Bruker, 2005 ▶); data reduction: *SAINT*; program(s) used to solve structure: *SHELXS97* (Sheldrick, 2008 ▶); program(s) used to refine structure: *SHELXL97* (Sheldrick, 2008 ▶); molecular graphics: *X-SEED* (Barbour, 2001 ▶); software used to prepare material for publication: *publCIF* (Westrip, 2010 ▶).

## Supplementary Material

Crystal structure: contains datablocks global, I. DOI: 10.1107/S1600536811000717/hg2789sup1.cif

Structure factors: contains datablocks I. DOI: 10.1107/S1600536811000717/hg2789Isup2.hkl

Additional supplementary materials:  crystallographic information; 3D view; checkCIF report

## Figures and Tables

**Fig. 1.**
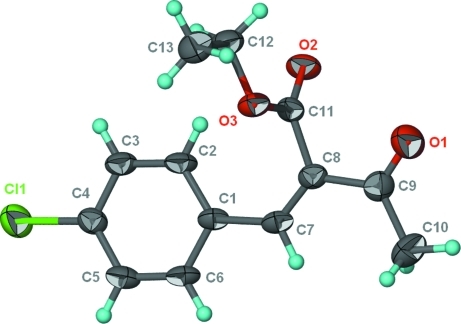
Thermal ellipsoid plot (Barbour, 2001) of C13H13ClO3 at the 50% probability level; hydrogen atoms are drawn as spheres of arbitrary radius.

## supplementary crystallographic information

### Comment

Trizma is a mildly-basic primary aminoalcohol that catalyzes Knoevenagel condensation reactions. The yield can be high under microwave irradiation; the title compound has been synthesized albeit by a conventional route. The carbon-carbon double-bond in C_13_H_13_ClO_3_ has a *Z*-configuration. The aliphatic substituents at one end of the double-bond, *i.e.*, the CH_3_CO– and planar C_2_H_5_O_2_C– groups, are aligned at 82.1 (3) ° with respect to each other. Bond dimensions in the molecule compare favorably with those found in similar molecules (Deng *et al.*, 2007; Shi, 2008).

### Experimental

Trizma (0.01 mol), *p*-chlorobenzaldehyde (0.01 mol) and ethyl acetoacetate (0.02 mol) were heated in ethanol (50 ml) for 3 h. The reaction was monitored with TLC. The solid that separated was collected and recrystallized from ethanol to give a colorless crystals, m.p. 373 K (60% yield).

### Refinement

Carbon-bound H-atoms were placed in calculated positions [C–H 0.93 to 0.97 Å; *U*(H) 1.2 to 1.5*U*(C)] and were included in the refinement in the riding model approximation, with *U*(H) set to 1.2 to 1.5*U*(C).

### Crystal data

**Table:**

C_13_H_13_ClO_3_	*F*(000) = 528
*M**_r_* = 252.68	*D*_x_ = 1.351 Mg m^−^^3^
Monoclinic, *P*2_1_/*n*	Mo *K*α radiation, λ = 0.71073 Å
Hall symbol: -P 2yn	Cell parameters from 3560 reflections
*a* = 9.9956 (6) Å	θ = 2.2–27.8°
*b* = 7.7487 (5) Å	µ = 0.30 mm^−^^1^
*c* = 16.2709 (10) Å	*T* = 295 K
β = 99.624 (1)°	Prism, colorless
*V* = 1242.49 (13) Å^3^	0.20 × 0.20 × 0.20 mm
*Z* = 4	

### Data collection

**Table:**

Bruker APEXII diffractometer	2790 independent reflections
Radiation source: fine-focus sealed tube	1968 reflections with *I* > 2σ(*I*)
graphite	*R*_int_ = 0.069
φ and ω scans	θ_max_ = 27.5°, θ_min_ = 2.2°
Absorption correction: multi-scan (*SADABS*; Sheldrick, 1996)	*h* = −12→12
*T*_min_ = 0.942, *T*_max_ = 0.942	*k* = −10→9
11255 measured reflections	*l* = −21→21

### Refinement

**Table:**

Refinement on *F*^2^	Primary atom site location: structure-invariant direct methods
Least-squares matrix: full	Secondary atom site location: difference Fourier map
*R*[*F*^2^ > 2σ(*F*^2^)] = 0.065	Hydrogen site location: inferred from neighbouring sites
*wR*(*F*^2^) = 0.201	H-atom parameters constrained
*S* = 1.02	*w* = 1/[σ^2^(*F*_o_^2^) + (0.1314*P*)^2^] where *P* = (*F*_o_^2^ + 2*F*_c_^2^)/3
2790 reflections	(Δ/σ)_max_ = 0.001
156 parameters	Δρ_max_ = 0.40 e Å^−^^3^
0 restraints	Δρ_min_ = −0.52 e Å^−^^3^

### Fractional atomic coordinates and isotropic or equivalent isotropic displacement parameters (Å^2^)

**Table:**

	*x*	*y*	*z*	*U*_iso_*/*U*_eq_	
Cl1	0.17886 (7)	0.43778 (11)	0.15024 (4)	0.0663 (3)	
O1	0.2270 (2)	0.3753 (3)	0.72305 (11)	0.0703 (6)	
O2	0.42363 (15)	0.4336 (2)	0.59728 (11)	0.0555 (5)	
O3	0.27248 (13)	0.6486 (2)	0.56982 (10)	0.0452 (4)	
C1	0.13562 (19)	0.3488 (3)	0.41918 (13)	0.0399 (5)	
C2	0.2540 (2)	0.4199 (3)	0.39776 (14)	0.0453 (6)	
H2A	0.3252	0.4497	0.4397	0.054*	
C3	0.2669 (2)	0.4463 (3)	0.31626 (14)	0.0467 (6)	
H3	0.3460	0.4940	0.3031	0.056*	
C4	0.1611 (2)	0.4015 (3)	0.25368 (14)	0.0437 (5)	
C5	0.0440 (2)	0.3274 (3)	0.27150 (14)	0.0490 (6)	
H5	−0.0258	0.2957	0.2290	0.059*	
C6	0.03270 (19)	0.3015 (3)	0.35374 (14)	0.0453 (5)	
H6	−0.0458	0.2509	0.3662	0.054*	
C7	0.11396 (19)	0.3175 (3)	0.50496 (14)	0.0418 (5)	
H7	0.0387	0.2501	0.5098	0.050*	
C8	0.18613 (19)	0.3714 (3)	0.57758 (13)	0.0405 (5)	
C9	0.1549 (2)	0.3219 (3)	0.66085 (15)	0.0501 (6)	
C10	0.0339 (3)	0.2111 (4)	0.66738 (18)	0.0693 (8)	
H10A	0.0333	0.1832	0.7248	0.104*	
H10B	0.0385	0.1068	0.6362	0.104*	
H10C	−0.0476	0.2727	0.6453	0.104*	
C11	0.3088 (2)	0.4853 (3)	0.58325 (13)	0.0395 (5)	
C12	0.3811 (2)	0.7742 (3)	0.57098 (17)	0.0535 (6)	
H12A	0.4275	0.7563	0.5239	0.064*	
H12B	0.4466	0.7634	0.6219	0.064*	
C13	0.3162 (3)	0.9486 (3)	0.56615 (18)	0.0592 (7)	
H13A	0.3836	1.0353	0.5626	0.089*	
H13B	0.2764	0.9679	0.6151	0.089*	
H13C	0.2470	0.9546	0.5176	0.089*	

### Atomic displacement parameters (Å^2^)

**Table:**

	*U*^11^	*U*^22^	*U*^33^	*U*^12^	*U*^13^	*U*^23^
Cl1	0.0540 (4)	0.0967 (7)	0.0483 (4)	0.0031 (3)	0.0085 (3)	0.0024 (3)
O1	0.0600 (11)	0.0996 (17)	0.0513 (11)	−0.0032 (11)	0.0095 (9)	0.0006 (10)
O2	0.0281 (8)	0.0621 (12)	0.0735 (12)	0.0041 (7)	−0.0001 (7)	0.0076 (8)
O3	0.0282 (7)	0.0479 (10)	0.0601 (9)	−0.0011 (6)	0.0091 (6)	−0.0004 (7)
C1	0.0237 (8)	0.0445 (12)	0.0514 (12)	−0.0006 (8)	0.0061 (8)	−0.0051 (9)
C2	0.0235 (9)	0.0614 (15)	0.0504 (12)	−0.0061 (9)	0.0049 (8)	−0.0091 (10)
C3	0.0280 (10)	0.0594 (15)	0.0533 (13)	−0.0052 (9)	0.0086 (9)	−0.0040 (10)
C4	0.0335 (10)	0.0524 (13)	0.0446 (11)	0.0046 (9)	0.0050 (8)	−0.0025 (10)
C5	0.0295 (10)	0.0605 (14)	0.0540 (13)	−0.0031 (10)	−0.0013 (9)	−0.0107 (11)
C6	0.0237 (9)	0.0528 (13)	0.0590 (13)	−0.0075 (9)	0.0060 (8)	−0.0092 (10)
C7	0.0246 (9)	0.0464 (12)	0.0555 (12)	−0.0016 (8)	0.0099 (8)	−0.0030 (10)
C8	0.0280 (9)	0.0422 (12)	0.0522 (12)	0.0038 (9)	0.0092 (8)	0.0033 (9)
C9	0.0405 (11)	0.0575 (15)	0.0545 (13)	0.0068 (10)	0.0147 (10)	0.0040 (11)
C10	0.0592 (16)	0.080 (2)	0.0759 (18)	−0.0097 (14)	0.0314 (14)	0.0065 (15)
C11	0.0284 (9)	0.0499 (13)	0.0400 (10)	0.0034 (9)	0.0046 (8)	0.0021 (9)
C12	0.0379 (11)	0.0576 (16)	0.0653 (14)	−0.0090 (10)	0.0092 (10)	0.0009 (12)
C13	0.0581 (16)	0.0522 (15)	0.0686 (17)	−0.0050 (12)	0.0140 (13)	−0.0020 (12)

### Geometric parameters (Å, °)

**Table:**

Cl1—C4	1.745 (2)	C6—H6	0.9300
O1—C9	1.213 (3)	C7—C8	1.344 (3)
O2—C11	1.201 (2)	C7—H7	0.9300
O3—C11	1.325 (3)	C8—C9	1.491 (3)
O3—C12	1.456 (3)	C8—C11	1.501 (3)
C1—C6	1.400 (3)	C9—C10	1.502 (4)
C1—C2	1.401 (3)	C10—H10A	0.9600
C1—C7	1.468 (3)	C10—H10B	0.9600
C2—C3	1.369 (3)	C10—H10C	0.9600
C2—H2A	0.9300	C12—C13	1.495 (4)
C3—C4	1.384 (3)	C12—H12A	0.9700
C3—H3	0.9300	C12—H12B	0.9700
C4—C5	1.377 (3)	C13—H13A	0.9600
C5—C6	1.376 (3)	C13—H13B	0.9600
C5—H5	0.9300	C13—H13C	0.9600
			
C11—O3—C12	116.98 (16)	O1—C9—C8	119.1 (2)
C6—C1—C2	117.2 (2)	O1—C9—C10	120.6 (2)
C6—C1—C7	118.26 (18)	C8—C9—C10	120.3 (2)
C2—C1—C7	124.50 (19)	C9—C10—H10A	109.5
C3—C2—C1	121.34 (19)	C9—C10—H10B	109.5
C3—C2—H2A	119.3	H10A—C10—H10B	109.5
C1—C2—H2A	119.3	C9—C10—H10C	109.5
C2—C3—C4	119.4 (2)	H10A—C10—H10C	109.5
C2—C3—H3	120.3	H10B—C10—H10C	109.5
C4—C3—H3	120.3	O2—C11—O3	125.2 (2)
C5—C4—C3	121.4 (2)	O2—C11—C8	124.1 (2)
C5—C4—Cl1	119.85 (17)	O3—C11—C8	110.72 (16)
C3—C4—Cl1	118.72 (18)	O3—C12—C13	106.71 (18)
C6—C5—C4	118.5 (2)	O3—C12—H12A	110.4
C6—C5—H5	120.8	C13—C12—H12A	110.4
C4—C5—H5	120.8	O3—C12—H12B	110.4
C5—C6—C1	122.12 (19)	C13—C12—H12B	110.4
C5—C6—H6	118.9	H12A—C12—H12B	108.6
C1—C6—H6	118.9	C12—C13—H13A	109.5
C8—C7—C1	130.0 (2)	C12—C13—H13B	109.5
C8—C7—H7	115.0	H13A—C13—H13B	109.5
C1—C7—H7	115.0	C12—C13—H13C	109.5
C7—C8—C9	123.8 (2)	H13A—C13—H13C	109.5
C7—C8—C11	123.3 (2)	H13B—C13—H13C	109.5
C9—C8—C11	112.85 (19)		
			
C6—C1—C2—C3	1.7 (3)	C1—C7—C8—C11	1.8 (4)
C7—C1—C2—C3	−179.6 (2)	C7—C8—C9—O1	179.1 (2)
C1—C2—C3—C4	−0.2 (4)	C11—C8—C9—O1	0.2 (3)
C2—C3—C4—C5	−1.3 (4)	C7—C8—C9—C10	−2.4 (4)
C2—C3—C4—Cl1	179.48 (18)	C11—C8—C9—C10	178.7 (2)
C3—C4—C5—C6	1.2 (4)	C12—O3—C11—O2	1.0 (3)
Cl1—C4—C5—C6	−179.59 (18)	C12—O3—C11—C8	−178.29 (18)
C4—C5—C6—C1	0.4 (4)	C7—C8—C11—O2	−99.3 (3)
C2—C1—C6—C5	−1.8 (3)	C9—C8—C11—O2	79.6 (3)
C7—C1—C6—C5	179.4 (2)	C7—C8—C11—O3	80.1 (3)
C6—C1—C7—C8	−169.6 (2)	C9—C8—C11—O3	−101.1 (2)
C2—C1—C7—C8	11.7 (4)	C11—O3—C12—C13	−172.43 (19)
C1—C7—C8—C9	−177.0 (2)		
